# How do you design randomised trials for smaller populations? A framework

**DOI:** 10.1186/s12916-016-0722-3

**Published:** 2016-11-25

**Authors:** Mahesh K. B. Parmar, Matthew R. Sydes, Tim P. Morris

**Affiliations:** 1 London Hub for Trials Methodology Research, MRC Clinical Trials Unit at UCL, Aviation House, 125 Kingsway, London, WC2B 6NH UK; 2 Medical Statistics Dept., London School of Hygiene and Tropical Medicine, Keppel Street, London, WC1E 7HT UK

**Keywords:** Randomised trials, Trial design, Smaller populations, Uncommon diseases

## Abstract

How should we approach trial design when we can get some, but not all, of the way to the numbers required for a randomised phase III trial?

We present an ordered framework for designing randomised trials to address the problem when the ideal sample size is considered larger than the number of participants that can be recruited in a reasonable time frame. Staying with the frequentist approach that is well accepted and understood in large trials, we propose a framework that includes small alterations to the design parameters. These aim to increase the numbers achievable and also potentially reduce the sample size target. The first step should always be to attempt to extend collaborations, consider broadening eligibility criteria and increase the accrual time or follow-up time. The second set of ordered considerations are the choice of research arm, outcome measures, power and target effect. If the revised design is still not feasible, in the third step we propose moving from two- to one-sided significance tests, changing the type I error rate, using covariate information at the design stage, re-randomising patients and borrowing external information.

We discuss the benefits of some of these possible changes and warn against others. We illustrate, with a worked example based on the Euramos-1 trial, the application of this framework in designing a trial that is feasible, while still providing a good evidence base to evaluate a research treatment.

This framework would allow appropriate evaluation of treatments when large-scale phase III trials are not possible, but where the need for high-quality randomised data is as pressing as it is for common diseases.

## Introduction

The design of phase III randomised controlled trials (RCTs) requires some estimate of the number of patients needed to answer a question of principal substantive interest. Sometimes there may be a discrepancy between this number and the number of patients who can be recruited in a reasonable time frame. This discrepancy may be small or very large. It may because the condition is uncommon, because the focus is on an uncommon subset of a common condition (e.g. through genetic stratification) or because outcomes for the patient group are already very good, meaning that events are uncommon and new treatments can only plausibly offer a small improvement. With increasing stratification of diseases into subdiseases, this problem of *too few patients for the feasible time* will only increase and there is a pressing need for high-quality trials to be run in these smaller populations.

**Fig. 1:**
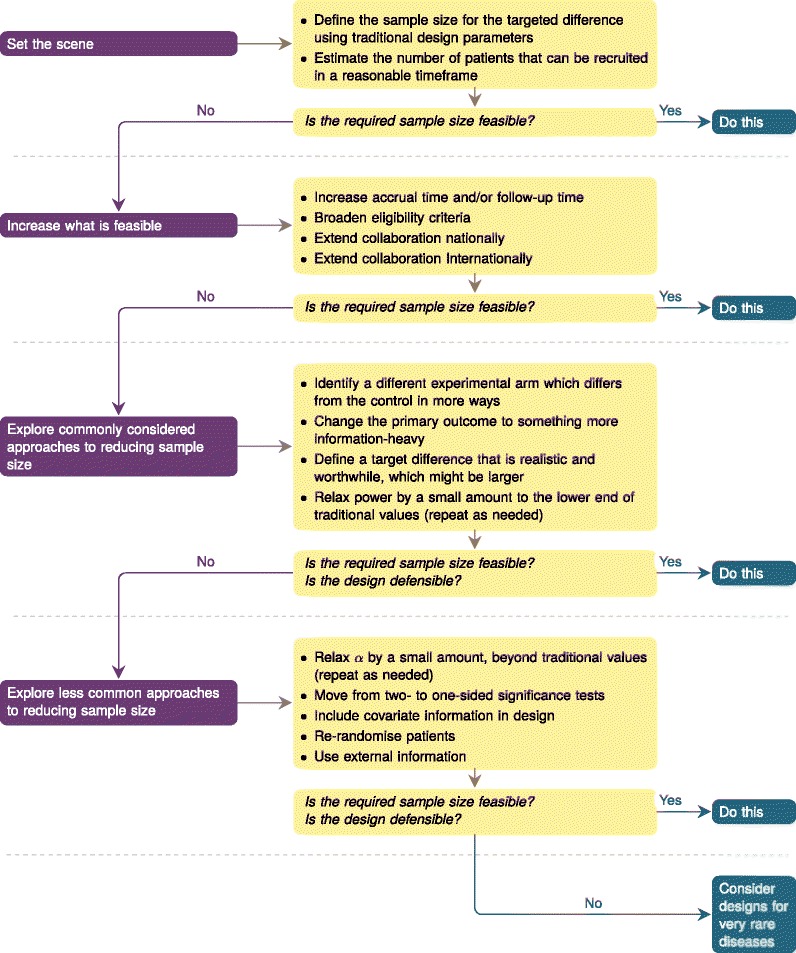
The framework for designing trials in smaller populations. Readers should use the corresponding subheadings in the text to understand the considerations for each element, particularly regarding context

How should we approach the design of high-quality RCTs in such settings? This paper provides a systematic approach to clinical trial design. We outline a framework that sets out a series of considerations for the team designing a trial. It encourages a period of constructive deliberation about the various design elements, each of which is carefully reconsidered. A simple depiction of the framework is given in Fig. 1. Some approaches aim to make the target sample size more achievable; others aim to make the target sample size smaller. Arguably any one of the alterations to the working design will change the character of the trial somewhat. Having considered all elements, it is up to the trial designer to decide whether this is preferable to not conducting a randomised trial.

This framework represents a systematic and structured approach to attacking this problem. It is not exhaustive or definitive and the elements considered are not new, though some of the contextual arguments are novel. This is intentional to ensure that the ideas are widely known and accepted. We also stress that changing some aspects of the design may alter the research question to a small or large extent. This will be made clear as each element is discussed. The extent to which the research question is changed will depend on the element and the disease context.

## Case study: the EURAMOS-1 trial

Osteosarcoma is a rare disease by any definition [1], with 150–200 new cases per year in the UK. The EURAMOS-1 protocol consisted of two RCTs designed to assess the effect of post-operative therapy on care. All patients underwent a three-drug induction regimen, consisting of two cycles of cisplatin and doxorubicin along with four cycles of methotrexate (MAP), before proceeding to surgical resection. After an assessment of the histological response of the tumour to chemotherapy at the time of surgery, one randomised comparison was conducted in ‘poor responders’ and another in ‘good responders’ (an example of subsetting referred to in the introduction). Here, we focus on the good responders, who were randomised between further MAP chemotherapy and MAPifn (which involved adding pegylated interferon *α*-2b as maintenance therapy for approximately 18 months after MAP).

The sample size calculation used event-free survival as the primary outcome. It was assumed that about 70 % of good responders would experience events on the control arm by 3 years, and looked to detect a hazard ratio of 0.63 (a 10 % absolute improvement) with 80 % power, using a two-sided 5 % significance level. This required 147 events across the two arms, which would necessitate accrual of 576 good responders over 3.5 years for an answer after 5 years in total.

After outlining the framework, we will consider how it could have been applied to the design and analysis of EURAMOS-1.

## Methods: The framework

The first step of the framework (Fig. 1) is to work out an ideal sample size and consider whether it is feasible. If it is not, the following sections describe how to apply different elements of the framework and how to approach evaluating them.

Reviewers, funders, collaborators, patients and regulators at the beginning, and regulators, journal editors, readers and patients at the end, will all need to be persuaded that the chosen design was the best approach and any unusual features were necessary. All these parties need to be persuaded of the key problem that the condition is not common enough to deliver the trial that would traditionally be expected, and that all efforts were made to maximise the number of patients available and the information obtained from them. Trials often take many years to design and deliver. Putting in many *months* to get the design right from the outset should not be considered disproportionate in terms of time or effort.

An important premise is to understand what we term a societal perspective: the view from the society affected by a specific disease. The aim of RCTs should be to find a way to improve outcomes for future patients.

## Step 1: Increasing what is feasible

If the trial was originally envisaged as enrolling patients from a single centre, city, region or country, with specific eligibility criteria, and following patients up for a specific period, it is important to consider how broadening the collaboration, making the eligibility criteria more inclusive and extending the follow-up could help to make the trial feasible.

### Increasing accrual time and/or follow-up time

Extending the follow-up time typically can increase the information content from a randomised trial and is particularly relevant for time-to-event data, where the sample size is determined by the number of events, rather than the number of patients.

### Broadening eligibility criteria

Investigators should reconsider eligibility criteria, treatments etc. to see if more participants can be entered into the trial. If eligibility criteria can be broadened while retaining patient safety and applicability of the plausible effect, then doing so is a good step. The research question of the trial itself may be altered by doing so: the patient group for whom the RCT provides an internal result is broadened. This may impact in a number of ways on how the result generalises outside of a RCT.

### Extending collaboration nationally and internationally

We propose thinking about extended collaboration in two steps. First, considering national collaboration, and second international collaboration. National collaboration may prove challenging owing to differences in opinion with other potential investigators, for example regarding the current standard care and research question. These are important to overcome, and the earlier the better. If a new intervention shows a benefit, these investigators may be the people who need to be persuaded to adopt it, so their involvement in the trial could be important. Such investigators will also provide a critical sounding board and may input to improve the design.

International collaboration can pose even more logistical difficulties, as funding and regulations governing research vary greatly across countries and continents. Further, conceptual difficulties may arise if the standard of care is different across countries, as this makes ‘control’ a difficult concept. This may alter the research question, from comparison with a specific and well-defined standard of care, to comparison with a broader and less well-defined group.

In some situations, this may be logistically complicated, leading to designing trials for a meta-analysis, such as MRC OV05 and EORTC 55955 [2], which were operationally separate trials but were analysed together as one throughout—including at interim analyses. We note that collaboration is still necessary, but slight differences to protocols may lead to more heterogeneity and so this should be considered a second option to collaboration under one protocol.

Some disease areas have acknowledged the difficulties with doing large-scale, high-quality academic and commercial randomised trials and have formed collaborative research networks. UK examples at the national level include the UK Dermatology Clinical Trials Network and the National Cancer Research Network; international examples are the Tuberculosis Trials Consortium, the International Rare Cancers Initiative [3] and the European Networks of Reference for Rare Diseases. These resources make it easier to approach collaborators and centres, which may increase recruitment.

## Step 2: Exploring commonly considered approaches to reducing sample size

Once the trial has been broadened as far as it can be, the next step must be to demonstrate to relevant stakeholders that the sample size requirement remains unachievable.

At this stage, several statistical aspects of the working design can be examined and potentially changed or adapted within the standard frequentist RCT paradigm. It is important to consider the implications for each trial individually. Any of these adaptations may be used in certain trials, but some come at a cost, or would only be useful or justified in some settings. The consequences of changes to the working design need to be thought through with care and clearly understood.

### Identifying a different experimental arm that differs from the control in more ways

The more similar the research and control treatments are, the less likely it is that the trial will show a difference in disease outcomes between arms. To quote from Peto et al. [4]: ‘Treatments must be sufficiently different from each other for it to be medically plausible that the death rate (or the rate of whatever type of event is of chief interest) on one could well be very substantially lower than on the other.’ If there are two or more candidate research treatments and only one can reasonably be tested, the treatment chosen for testing should be as different as possible to the standard to maximise the chance of showing a difference. Furthermore, it would be inefficient or even wasteful to spend time looking at small differences or non-inferiority questions in settings where there are very few opportunities to improve outcomes.

Changing the experimental arm in this way can mean that the research question is changed. This may be regarded as justifiable from a societal perspective when there is no commitment to a specific treatment; only a commitment to pursue the one which offers the best opportunity to improve outcomes.

When a candidate intervention is made up of multiple components and the context means a factorial design is infeasible, this advice suggests changing multiple components at once for the intervention arm. This, of course, carries a risk of being unable to identify which components are effective. However, failing to make the intervention arm different enough to the control—and thus failing to improve outcomes for patients—is arguably a bigger risk.

### Changing the primary outcome to something more information heavy

Statistically speaking, the best primary outcome to use is the one with the greatest information content; that is, the one which minimises the variance of the treatment effect relative to the magnitude of the treatment effect. In terms of information content, there is generally a hierarchy for outcome measures with continuous outcomes tending to hold most information, followed by, in order, time-to-event, ordinal and finally binary outcome measures. From a statistical perspective, it is, thus, sensible to use the most information-rich primary outcome available. It is always costly in terms of sample size to split continuous or ordered outcome data into two categories.

Clearly the primary outcome measure must be important from the perspective of both patients and treating clinicians: the practical convenience of needing fewer patients should not determine the choice of outcome unless candidate outcome measures are considered relevant for decision-making for all interested parties, including patients, clinicians, relevant health authorities and, potentially, regulators.

It is also important to consider any other studies in the field or closely related areas, so that common outcome measures might be measured in all studies to facilitate the synthesis of evidence.

### Defining a target difference that is realistic and worthwhile

The target difference between groups is often selected as being both (1) realistic and (2) large enough that it is to be likely to be important to the patient and the clinician. If such a target difference cannot be agreed upon, then the value of the research treatment as a trial candidate should be questioned.

Methods for specifying a target difference and guidance for doing so are given in [5]. How can a *realistic* difference be identified? In looking for answers, judicious use of existing data can be very helpful. It is worth looking at as many related trials as possible for information. In particular, one might consider, in an approximately hierarchical order:

- The same therapy in other diseases
- Related therapies in the same disease
- Studies with this therapy in different disease stages (and possibly earlier phases) in the same disease (acknowledging the potential for over-optimism)
- More generally, previous studies of other therapies in the same and other diseases

Earlier phase trials need more care, as they often include continuous outcome measures that can be assessed quickly but in most diseases these measures may have less direct relevance to the long-term outcomes. Additional information is then needed on the association between this early-phase outcome and the proposed outcome for the trial at hand to identify plausible target differences for the proposed outcome.

It is possible to reduce the required sample size by increasing the targeted treatment effect and sometimes this process leads to targeting an effect that can be detected given the numbers that can be recruited [6]. An increased targeted treatment difference must have plausibility and, if possible, a clinical evidence base. If there is no such basis, designers should be aware that the power to detect a more realistic effect that might still be deemed worthwhile is low and unclear (although it should always be explicitly calculated). Specifying a large, unrealistic difference is effectively low power for a realistic difference in disguise.

### Relaxing power by a small amount to the lower end of traditional values

Researchers should consider the question: ‘What are the consequences of [erroneously] deciding *not* to use a new treatment that is truly better?’

For some treatments and diseases, the cost (not just economic) of missing a good treatment may be quite high. We will argue that answers may be rather different in smaller population settings, compared to very common diseases.

To understand our arguments, consider the societal perspective when we are looking for a randomised trial to *improve outcomes*. Whether or not this is through the specific intervention under study in a given trial or some other intervention is of little interest. When a disease is experienced by very many patients, there may be many trials evaluating many different interventions. However, in diseases affecting smaller populations, from which it is hard to recruit patients, although there may be many new interventions appearing, opportunities to evaluate all, or indeed many, will not be possible.

To illustrate this point, we searched through PubMed and ClinicalTrials.gov (the search was made on 10 December 2015) for registered trials in arbitrarily chosen diseases with many potential patients for trials (breast, lung and colorectal cancers, and asthma), and in diseases we would expect to have a shortage of available patients for trials at any one time (osteosarcoma and muscular dystrophy). The search was for phase III RCTs added (clinicaltrials.gov) or published (PubMed) after 1 January 2015. The specific search terms and results are included as supplementary material.

The results of our search are given in Table 1. It is clear that in breast, lung and colorectal cancer, and in asthma, there are many trials and, therefore, many potential chances to improve outcomes. Reducing power means that fewer patients are required, but makes discovery of an effective treatment less likely. Trials typically opt for 90, 85 or, at the lowest, 80 % power, the latter giving a 1 in 5 chance of missing that a treatment really works (this relates to the arguments of Ioannidis [7]).

**Table 1:** Number of trials published or added between 1 January 2012 and 15 December 2015

				Expected hypothetical		
				false positives^a^		
Category	Disease	PMC	ct.gov	(PMC | ct.gov)		
				40 %	10 %	70 %
Larger	Asthma	216	110	4 | 2	1 | 1	6 | 3
Populations	Breast cancer	632	897	13 | 18	3 | 4	19 | 27
	Colorectal cancer	470	122	9 | 2	2 | 1	14 | 4
	Lung cancer	380	149	8 | 3	2 | 1	11 | 4
Smaller	Muscular dystrophy	9	11	0 | 0	0 | 0	0 | 0
Populations	Osteosarcoma	8	2	0 | 0	0 | 0	0 | 0

*PMC* PubMed Central, *ct.gov* ClinicalTrials.gov

^a^Assuming 40 %, 10 % and 70 % positive results in each area and a 5 % type I error rate

Consider the societal perspective relating to studies in a particular disease. The chance of improving outcomes for future patients increases quickly when there are more trials running. If three trials each achieve just 50 % power to detect a difference when testing truly effective interventions, the power to detect a difference in one or more of these trials (and thus achieve the societal aim of improving future patients’ outcomes) is 87.5 %. If three trials each have 80 % power individually, the joint power becomes 99.2 %. However, with just one trial with 80 % power, this represents all the power to improve outcomes in an area.

When the trial being designed may be the only chance of improving outcomes in 5 or 10 years, a single trial may form most or all of the evidence base. It is paradoxically more important to have good power in this individual trial in a smaller population than in conditions where multiple randomised trials can be run simultaneously. The opportunity cost of a false negative is greater because the opportunity to interested parties is more precious. We advise researchers to avoid compromising on power in these smaller population settings, because other trials that can improve outcomes for this patient group are unlikely to emerge.

## Exploring less common approaches to reducing sample size

We now consider some less standard approaches to bringing the sample size requirements closer to the numbers it is feasible to recruit in a reasonable time frame.

### Step 3: Relaxing *α* by a small amount, beyond traditional values

The much-criticised 5 % significance level is used widely in much applied scientific research, but is an arbitrary figure. It is extremely rare for clinical trials to use any other level. It may be argued that this convention has been adopted as a compromise between erroneously concluding a new treatment is more efficacious and undertaking a trial of an achievable size and length. Settings where traditionally sized trials are not possible may be just the area where researchers start to break this convention, for good reason.

In considering the type I error, it is critical to consider the question: ‘What are the consequences of erroneously deciding to use a new treatment routinely if it is truly not better?’

Taking the societal perspective as before, we might consider the probability of making a type I error, thus erroneously burdening patients with treatments that do not improve outcomes, or even worsen them, while potentially imposing unnecessary toxicity.

First, for conditions where there are only enough patients available to run one modestly sized randomised trial in a reasonable time frame, research progress will be relatively slow, and making a type I error may be less of a concern than a type II error. In contrast, making several type I errors in a common disease could lead in practice to patients taking several ineffective treatments; for a disease area where only one trial can run at any given time, the overall burden on patients is potentially taking one ineffective treatment that does not work.

Thus, if we take the societal perspective with the trials in Table 1 then, if each trial was analysed with *α*=0.05 and we see (hypothetically) 40 % positive results [8], then the expected number of false positive trials is given in the final column. We also assumed 10 % and 70 % positive results, with qualitatively similar conclusions.

It is worth viewing this as a consideration of the joint type I error rate of these trials. If there are *t* trials published claiming a positive result, each specifying *α*=0.05, then the chance that a type I error will be made equals 1−(1−0.05)^*t*^. If *t*=1 as we are considering, the type I error rate equals 5 %. This increases to 14.3 % if three trials return a positive result. From a societal perspective, such an error rate may be more important than the 5 % levels specified in individual trials, a rarely acknowledged consideration.

When a new intervention has some toxicity, this argument requires even greater consideration. Assume the intervention is in truth not better (possibly worse) than the control arm and returns a high but tolerable level of toxicity. If it is falsely judged to be superior in a trial (i.e. a type I error is made), there are implications for future research. Patients will already be experiencing some ln, narrowing the path for future treatment options, particularly if a future RCT is one of adding a treatment rather than substituting: any further toxicity may make the total toxicity unacceptable. In this case, significance levels (and target differences) should be chosen with a clear consideration of the likely toxicity.

We note recent work that highlights the importance of considering long-term aims or research in context [9]. Rather than simply setting error rates for a single trial, one might consider a long-term horizon and the aims by that point. For example, running several smaller trials with relaxed *α* levels may lead to improved expected survival in the long term vs. fewer large trials with more stringent *α*, though more type I errors will be made (see [9] for details).

### Moving from two- to one-sided significance tests

Two-sided tests look for a difference between groups, but are technically agnostic about the direction of this difference. So-called superiority randomised trials aim to show that one treatment is superior to another on major disease outcomes. Two-sided testing is a ritual that involves careful neglect of the substantive hypothesis and it could be argued that it should be abandoned in most superiority RCTs [10].

In a trial looking to detect superiority of one treatment over another, a two-sided hypothesis test says we will reject *H*_0_: difference = zero if the new treatment is better or worse. However, if a two-sided test returns a *p* value of 0.0001 and the new treatment is worse, the decision would be not to use that treatment. The same decision would be made if the *p* value were 1 and the difference between treatments 0. There is, thus, a disconnect between the statistical hypothesis tests and the operational hypothesis interpretation. Note that a trial that finishes with a highly statistically significant value against the research arm is wasteful and harmful; pre-planned interim analyses should have been used to get out early, and to focus the limited resources into a randomised trial that tested something that might make a difference. Operationally, for superiority trials, both the hypothesis we are primarily interested in and its interpretation are very one-sided (‘harm’ and ‘no-effect’ lead to one decision, while ‘benefit’ leads to another). Researchers using a nominally two-sided, 5 % significance level are effectively using a one-sided, 2.5 % significance level. To improve efficiency, the statistical design could better reflect this behaviour, and employ one-sided testing procedures and intervals.

In many sequential or adaptive designs, it is already common to design and analyse with one-sided significance levels because decisions may otherwise be nonsensical, for example in designs that aim to stop for futility [11].

Note that this argument is not related to the societal perspective adopted in arguing for high power and higher type I error rates than conventionally used.

**Fig. 2:**
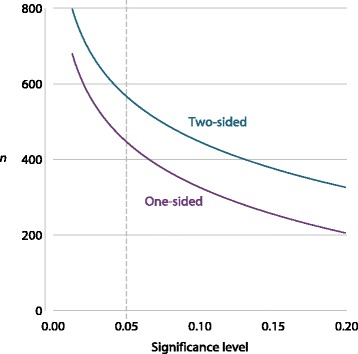
Dependence of required number of patients on sidedness of tests and desired type I error rate

Figure 2 shows how the target number of patients for EURAMOS-1 depends on the sidedness of tests and on the significance level chosen, with all other design parameters held constant.

### Including covariate information in design

Covariates are patient characteristics measured at baseline. In observational studies, adjusting for covariates can reduce bias due to confounding. In randomised trials, accounting for covariates has a different aim: to increase precision, and thus gain power [12, 13].

Adjusting the treatment effect estimate for covariates that affect the outcome measure has been shown to lead to substantial increases in power [14]; adjusting for covariates that do not affect the outcome measure leads to a slight loss, but this is very small [14]. There are several alternative methods of accounting for covariates [13, 15, 16].

When trying to compute sample size requirements, it is possible in principle to allow for covariates. For continuous outcome measures, this may be through reducing the standard deviation in sample size calculations (because covariate effects will explain some of the variation away). However, it is not clear how best to approach this for categorical or time-to-event outcome measures, and is an area worthy of methodological research.

Previous work based on 12 different outcomes taken from eight studies demonstrated that the effects of covariates seen in real trials could increase power from 80 % without covariate adjustment to between 81 and 99 % with planned covariate adjustment [14]. Without formally incorporating covariate effects in the sample size calculations, planning to adjust the analysis may be viewed as a method of reclaiming some power. Compared to the sample size calculations, we may expect this to be in the region of about 5 % (and hope it is more). This may be a way of strengthening the design if power has been relaxed further than we would wish.

### Re-randomising patients

Historically, the only context in which patients are permitted to participate in the same trial more than once is in crossover designs, which involve patients being randomly assigned to a sequence of treatments and having outcomes measured after each period, with some or all patients receiving different treatments in different periods (crossing over) [17]. A predefined number of treatment periods is set out for each patient.

However, re-randomising patients who have completed their predefined follow-up from a previous randomisation in the trial and who continue to meet the trial’s eligibility criteria an arbitrary number of times can still result in valid statistical inference about treatment effectiveness [18]. Unlike crossover trials, patients do not have a predefined number of treatment periods and the treatment assignments in the sequence do not depend on previous or subsequent assignments.

The design will be suitable for diseases for which treatments are given repeatedly and follow-up is not long term. For example, it has been used for the treatment of febrile neutropenia and sickle-cell crises. It will be unsuitable in some settings: where long-term follow-up is required (particularly for economic evaluations), where the effectiveness of treatment depends on whether and how much it has previously been received (such as where the intervention is educational) or where a period of treatment and follow-up would mean patients are no longer eligible (for example where the primary outcome measure represents a move into a different disease state). Re-randomisation would be unsuitable for treatment of cancers when prolonged follow-up is required or when a procedure can only occur once, such as appendicectomy.

When patients require regular repeated treatments and outcomes are relatively short term, re-randomisation may inject extra numbers without having to compromise on other aspects of the design. If the majority of patients are randomised on multiple occasions then the analysis can be based on within-patient comparisons, potentially gaining much efficiency [18].

### Using external information

A treatment that works in one category of a broadly defined disease may work in related categories. That is, it is plausible that a treatment that works well in one specific disease category would have similar effects in another, even if it is unlikely that the effect will be exactly the same. Chemoradiotherapy is effective in three squamous cell carcinomas: head and neck, cervical and anal. It is therefore plausible that it would be effective in penile and vulval cancer, which are also squamous cell cancers but have far smaller patient populations. This may bolster the choice to relax *α* in that there is a precedent for the treatment working in closely related conditions, as it would be unlikely to have seen false positives in head and neck, cervical and anal cancer.

The notion of borrowed external information is particularly relevant for considering adverse events that are rare or only appear in the long term. Trials are rarely sized to specifically assess adverse events but it is critical that they are considered. If a treatment is indicated for other conditions then its adverse effects may already be reasonable well characterised, unless there are expected interactions with this specific patient group or another treatment with which the treatment under scrutiny has not previously been combined. In such a setting, a trial may be regarded as verifying the adverse-effect profile of treatment rather than demonstrating them for the first time.

External information on covariate effects can be particularly useful if the sample size will be calculated allowing for covariate effects.

Using external information does not impact on the research question, rather the information that will be brought to bear on that question. We do not aim here to prescribe how external information should be borrowed. However, Bayesian approaches lend themselves naturally to this problem and have been well explored [19]; formal frequentist approaches have been less well explored.

## Results: Applying the framework to EURAMOS-1

Following our framework, we can reconsider the design of EURAMOS-1.

Recall that the trial design required many hundreds of patients. No countries or single collaborative group could reasonably undertake such a trial alone within a decade. Therefore, the lead investigators spent many years developing a collaboration to run a joint protocol between four co-operative osteosarcoma groups across Europe and North America. This international collaboration considered it feasible to recruit 567 good responders over 3.5 years. This broad collaboration, which included discussion and compromise on key aspects, allowed a phase III trial to be undertaken [20, 21].

The trial maximised its collaboration and eligibility criteria, and investigated a research treatment quite different from the standard. The follow-up time could potentially be increased. The primary outcome measure was the most relevant and appropriate compared to all those that could have been used and no relevant alternative would provide more information. The target hazard ratio (HR) of 0.63 may be regarded as optimistic as we rarely see new treatments displaying this level of benefit in oncology (in surgically treated patients); we would begin by altering it to 0.7, which is probably more realistic and still worthwhile.

The two-sided 5 % significance level could be relaxed and changed to one-sided. Power was 80 % and we would avoid going any lower. Re-randomisation could not have been employed in EURAMOS-1 because only newly diagnosed patients were eligible; the relapse disease state, which might have been the point of re-randomisation, is different, thus such patients were not eligible to join at that point in their journey.

Table 2 shows the number of patients and events needed for sequential changes to the design. The actual design used is given in the first row. The second row changes the target HR to 0.7, and is the comparator when other changes are applied. First, one-sided tests are used. Second, the follow-up time is increased by 6 months. Finally, *α* is relaxed to 0.06, 0.07 and 0.08. By choosing the latter, these three changes alone mean we require 527 patients and 161 events, savings of 402 patients and 89 events compared with row 2.

**Table 2:** Using the framework to change the working design of EURAMOS-1

Scenario	Number to recruit	Events required	Time for:
	(change vs. row 2)	(change vs. row 2)	recruitment – follow-up
(Actual)	576	147	3.5 years – 5 years
Increase target HR to 0.7	929 (0)	250 (0)	3.5 years – 5.0 years
Extend follow-up by 6 months	820 (–109)	250 (0)	3.5 years – 5.5 years
Move to one-sided tests	646 (–283)	197 (–53)	3.5 years – 5.5 years
Relax alpha to:			
6 %	600 (–329)	183 (–67)	3.5 years – 5.5 years
7 %	561 (–368)	171 (–79)	3.5 years – 5.5 years
8 %	527 (–402)	161 (–89)	3.5 years – 5.5 years

This is like the original target and would be achievable through the international collaboration, with a robust and defensible design. For the primary analysis of EURAMOS-1, it was planned to use a Cox model adjusted for stratification factors (trial group, location of tumour and presence of metastases), which should increase the true power to 85 % or more.

## Discussion

The framework we have outlined represents an approach to systematic and structured thought about clinical trial design in settings where very large-scale trials are not feasible and is based on the frequentist paradigm that dominates in phase III RCTs. We advocate using approaches in Fig. 1 iteratively, making one change at a time through detailed discussions, and assessing whether the trial design is defensible and provides meaningful answers to a relevant research question.

This may result in one of four general paths:

1. Using a traditional trial with a broader scope than first envisaged
2. Using a traditional design with one or several of the alterations we have suggested considering
3. Opting for Bayesian designs for very rare diseases [22]
4. Deciding not to do a trial

Our proposed framework is based on pursuing (1) and (2) as far as possible. Some elements of our framework may alter the character of a given trial to the extent that the original research question is somewhat changed. It is important to be aware of this. If this is not desired, then the alternatives are (3) or (4). We reiterate the societal perspective that a trial should aim to improve outcomes for the patients affected. Alterations to elements of the design that are consistent with this aim should be considered, which may open the design to considerations.

The arguments for some elements are not unique to smaller populations and would also be worth considering in larger populations (for example, including covariates and re-randomising patients).

Because of the iterative nature of our approach to design, discussions with interested parties may take many months. This may appear to be a significant barrier to designing the trial. However, in our opinion, not enough time is generally devoted to designing individual trials. Forging ahead with a design that does not offer a proper opportunity to improve outcomes is failing the patients it aims to help, and the price is paid by the public and patients rather than the researchers. Therefore, we reiterate that adequate time should be set aside to get the design right.

Important work is ongoing on trials in extremely small populations where only very few individuals may feasibly be recruited [22, 23]. The EU have funded three projects to develop and improve design and analysis in these settings: *Inspire*: FP7 health 2013–602144; *Ideal*: FP7 health 2013–602552; and *Asterix*: FP7 health 2013–603160. Here, trial design and analysis necessarily requires a paradigm shift to less familiar designs, where the sample size target may be many times larger than the entire patient population; see for example [23, 24]. Bayesian methods may be used through necessity rather than choice and important design ideas, such as concurrent control groups and power, sometimes diminish or disappear.

We chose to adhere to the frequentist paradigm and retain the central concept or randomisation, which is most familiar to researchers, review bodies and regulators, because the majority of phase III trials employ a frequentist design and analyses. If Bayesian designs are being considered *only* because the frequentist design gives inadmissible numbers, then much of the information expected from a Bayesian design must be based on a prior. Exploring frequentist designs with this framework may thus offer a more satisfactory solution.

We have stressed throughout that the design elements researchers choose to alter will vary according to disease setting and the context of the trial. We regard accounting for covariates at the design stage as being an obvious choice with almost no cost. However, there is little practical guidance on how to do this, particularly in the context of survival data, and this is a topic worthy of research and guidance. Conversely, it is practically very simple to alter the target effect, power and type I error, but each of these comes with a clear cost.

Our aim in producing this framework is to bring systematic and structured thought to the design. We hope current and future methodology research will produce more elements to include in such structured thinking.

## Abbreviations

ASTERIX: Advances in small trials design for regulatory innovation and excellence
ct.gov: ClinicalTrials.gov
EU: European Union
Euramos: European and american osteosarcoma study
IDEAL: Integrated design and anaLysis of small population group trials
InSPiRe: Innovative methodology for small population research
MAP: methotrexate
MAPifn: methotrexate plus pegylated interferon *;1-2b*
PMC: PubMed Central
RCT: randomised controlled trial
UK: United Kingdom
